# Excess dietary Lys reduces feed intake, stimulates jejunal CCK secretion and alters essential and non-essential blood AA profile in pigs

**DOI:** 10.1186/s40104-023-00971-9

**Published:** 2024-02-18

**Authors:** Maximiliano Müller, Elout Van Liefferinge, Alan Tilbrook, Robert van Barneveld, Eugeni Roura

**Affiliations:** 1 Centre for Nutrition and Food Sciences, Queensland Alliance for Agriculture and Food Innovation, The University of Queensland, Brisbane, Queensland 4072 Australia; 2 Laboratory of Animal Nutrition and Animal Product Quality (LANUPRO), Department of Animal Sciences and Aquatic Ecology, Ghent University, 339000 Ghent, Flanders Belgium; 3 Centre for Animal Science, Queensland Alliance for Agriculture and Food Innovation and the School of Veterinary Science, The University of Queensland, Brisbane, Queensland 4072 Australia; 4 SunPork Group, Brisbane, Queensland 4009 Australia

**Keywords:** Amino acid, Blood, Cholecystokinin, Feed intake, Lysine, Pig, Satiety

## Abstract

**Background:**

Commercial diets are frequently formulated to meet or exceed nutrient levels including those of limiting essential amino acids (AA) covering potential individual variations within the herd. However, the provision of dietary excess of AA, such as Lys, may lead to reduced appetite and growth in pigs. The mechanisms modulating these responses have not been extensively investigated. This study evaluated the effect of Lys dietary excesses on performance and satiety biomarkers in post weaning pigs.

**Methods:**

Twenty-four pigs aged 21 d and weighing 6.81 ± 0.12 kg (mean ± SEM) were individually housed and offered 1 of 4 dietary treatments for 3 weeks: a diet containing a standardized ileal digestible Lys reaching 100% (T0), 120% (T1), 150% (T2) or 200% (T3) of the NRC (2012) requirements. At the end of the experiment, blood samples from the cephalic vein of the T0 and T3 groups were obtained for AA analysis. In addition, primary intestinal cultures from T0 pigs were used, following their humane killing, to evaluate the effect of Lys on gut hormone secretion and AA sensors gene expression under ex vivo conditions.

**Results:**

Feed intake was linearly reduced (*P* < 0.001) and the weight gain to feed ratio reduced (*P* < 0.10) with increased dietary levels of Lys during the third- and first-week post weaning, respectively. Cholecystokinin concentration (*P* < 0.05) and the metabotropic glutamate receptor 1 and the solute carrier family 7 member 2 (*P* < 0.10) gene expression was enhanced in proximal jejunum tissues incubated with Lys at 20 mmol/L when compared to the control (Lys 0 mmol/L). Plasma Lys and Glu (*P* < 0.05) concentration increased in the T3 compared to T0 pigs. In contrast, plasma levels of His, Val, Thr, Leu (*P* < 0.05) and Gln (*P* < 0.10) were lower in T3 than T0 pigs.

**Conclusion:**

The present results confirm that excess dietary Lys inhibits hunger in pigs. Moreover, the results provide evidence of pre- and post-absorptive mechanisms modulating these responses. Lys dietary excesses should be narrowed, when possible, to avoid negative effects of the AA on appetite in pigs.

## Background

Commercial pig feeds are frequently formulated to meet or exceed the requirements of key nutrients, such as limiting essential amino acids (AA). Nutritionists commonly apply margins above the minimum levels to account for potential practical issues (e.g., poor mixing) and potential individual variations on the nutritional requirements within the herd [1]. However, the excessive intake of some AA has been associated with reduced appetite in pigs [2]. Lys, the most limiting essential AA, can trigger satiation (feeling of fullness that leads to the termination of a meal) and satiety (feeling of fullness that prevents the initiation of a new meal) when provided in excess in pigs, such as through an oral gavage [3]. The negative effect of Lys on feed intake has been partly associated with the release of gut hormones with anorexigenic properties, such as cholecystokinin (CCK) [4]. While the release of CCK has been linked with the activation of enteroendocrine cells in the duodenum, previous studies in our group and others indicated that the effect of Lys on the release of CCK may occur mainly in the jejunum or other more distal segments of the gastrointestinal tract [2, 5]. In addition, the short half-life of CCK combined with the long-term effects of the AA on feed intake indicate that post absorptive mechanisms may also be involved in the satiating effect of Lys in pigs [3].

Following intestinal absorption into the portal vein and potential hepatic uptake, AA that reach the central nervous system (CNS) may function as appetite regulators, signalling the brain about the body metabolic status [6–8]. Reduced feed intake has been observed following the intake of dietary excesses of Leu in pigs [9]. The satiating effect triggered by the ingestion of high doses of Leu has been attributed for the most part to the detection of branched-chain amino acid (BCAA) imbalances in blood reaching the CNS [10, 11]. Changes in the circulating concentrations of essential amino acids (EAA), including BCAA, and non-essential amino acids (NEAA) have been noted following the ingestion of Lys excess diets in pigs [12, 13]. However, the blood kinetic of dietary Lys and the relationship with feed intake has not been extensively studied. A better understanding of the role of Lys on appetite modulation could help optimize feed formulations and enhance performance during the post weaning period, where low feed intake and body weight gain are frequently monitored in pigs.

The aim of the present study was to investigate the effect of dietary excess of Lys on performance and satiety related biomarkers including gut hormone secretion (CCK and glucagon-like peptide 1 (GLP-1)) and AA sensor gene expression in the gastrointestinal tract, as well as the blood kinetic of AA in post weaning pigs. It was hypothesized that dietary excess of Lys will reduce feed intake and increase the secretion of CCK in the proximal jejunum of post weaning pigs.

## Materials and methods

### Animals, housing, and diets

Twenty-four newly weaned (24 days of age) pigs (Landrace × Large White; body weight (mean ± SEM) = 6.81 ± 0.12 kg) were assigned to 4 dietary experimental groups with homogeneous body weight and balanced gender, and individually housed in slatted floor pens (1.7 m × 1.2 m) at the Herston Medical Research Centre at The University of Queensland (Herston Campus, Brisbane, Australia). Pigs were kept with 12 h of light (intensity of 40–60 lx) and the room temperature was maintained at 29 °C during the first week and then progressively decreased by 1 °C weekly. Pigs had ad libitum access to feed and water throughout the experiment. The 4 experimental diets were: a standard starter diet meeting 100% the requirements set by the NRC (1.20 standardised ileal digestible (SID) Lys; T0), or 3 dietary treatments with an excess SID Lys reaching 120% (T1), 150% (T2) or 200% (T3) of the NRC recommendations [14]. To differentiate the Lys effect on appetite from an increase in dietary nitrogen content, Ala (L-isoform, food grade) (Bulk Supplements, Henderson, USA) was supplemented to T0, T1 and T2 to make all diets isonitrogenous. Alanine was chosen over other potential AA based on its previously reported lack of impact on appetite and gut peptide secretion in pigs [2, 5]. The composition of experimental diets is shown in Table 1. Feed intake was measured daily and body weight weekly (d 7, 14 and 21).

**Table 1:** Composition of the experimental diets (as-fed basis)

Item	Dietary treatments^1^			
	T0	T1	T2	T3
Ingredients, %				
Wheat	70.61	70.61	70.61	70.61
Soyabean meal	8.60	8.60	8.60	8.60
Full fat soya	4.80	4.80	4.80	4.80
Meat bone meal	5.35	5.35	5.35	5.35
Fish meal	5.00	5.00	5.00	5.00
Vegetable oil	3.00	3.00	3.00	3.00
Lysine HCl	0.46	0.77	1.23	1.98
DL-Methionine	0.10	0.10	0.10	0.10
Threonine	0.15	0.15	0.15	0.15
Tryptophan	0.01	0.01	0.01	0.01
L-Alanine	1.45	1.16	0.72	–
Salt	0.20	0.20	0.20	0.20
Vitamin and mineral premix^2^	0.20	0.20	0.20	0.20
Calculated nutrient content, %				
Dry matter	90.61	90.61	90.61	90.61
Crude protein	20.60	20.60	20.60	20.60
Fat	6.10	6.10	6.10	6.10
Digestible energy, Mcal/kg	3.59	3.60	3.60	3.61
Calcium	0.96	0.96	0.96	0.96
Phosphorus	0.76	0.76	0.76	0.76
Lysine	1.29	1.53	1.88	2.48
SID Lys^3^	1.20	1.44	1.80	2.40
Methionine	0.42	0.42	0.42	0.42
Threonine	0.82	0.82	0.82	0.82
Tryptophan	0.22	0.22	0.22	0.22
Met/Lys	0.32	0.27	0.22	0.17
(Met + Cys)/Lys	0.56	0.47	0.38	0.29

^1^*T0* 100% Lys requirement, *T1* 120% Lys requirement, *T2* 150% Lys requirement, *T3* 200% Lys requirement based on its standard ileal digestibility

^2^Provided the following (as-fed basis): vitamin A (10,000 IU/kg), vitamin D_3_ (1,800 IU/kg), vitamin E (100 mg/kg), vitamin K_3_ (5 mg/kg), vitamin B_1_ (3 mg/kg), vitamin B_2_ (6 mg/kg), niacin (30 mg/kg), pantothenic acid (30 mg/kg), pyridoxine (4 mg/kg), biotin (0.3 mg/kg), folic acid (2.5 mg/kg), vitamin B_12_ (0.04 mg/kg), iron (100 mg/kg), iodine (0.7 mg/kg), manganese (45 mg/kg), selenium (0.3 mg/kg), zinc (120 mg/kg), cobalt (0.3 mg/kg), copper (10 mg/kg)

^3^Standard ileal digestible lysine

### Blood and tissue sample collection

On d 22, blood samples (2 mL) were collected from the right cephalic vein of T0 and T3 (control and highest Lys level, respectively) pigs. To facilitate the procedure (and minimize stress) pigs were moved to a separate room, anaesthetized with isoflurane (2%–3%) via mask ventilation and placed in the dorsal recumbency position on top of a surgical table with the right forelimb extended backward and slightly outward (to expose the cephalic vein). Upon collection, blood samples were immediately transferred into 2 mL EDTA vacutainers (Becton, Dickinson and Company, Franklin Lakes, USA) and placed on ice before further processing (i.e., centrifugation). Blood samples from the lowest and highest Lys treatments were chosen for collection to distinctly identify the AA blood profile related to the consumption of dietary Lys excesses in weaning pigs. Prior to blood collection, all animals had access to a morning meal for 30 min. All feeders were removed 1 h before blood sampling. The time of blood collection was chosen based on previous reports on AA blood kinetics in pigs [15].

Following the blood sampling, pigs were humanely killed using Lethabarb® (intravenous administration, 162.5 mg/kg) (Virbac, Milperra, Australia). The small intestine was then removed from the carcass, and the length measured from the pylorus to the ileocecal valve. Proximal jejunum (approx. 100–120 cm distal from the pylorus) and ileum (10 cm proximal from the ileocecal valve) from control pigs (T0 group) were immediately removed and placed in ice cold Krebs-Ringer Bicarbonate (KRB)/HEPES buffer adjusted to pH 7.4 bubbled with O_2_/CO_2_ (95%/5%) and promptly transported to the laboratory (The University of Queensland, St Lucia Campus, Brisbane, Australia) for the preparation of primary intestinal cell cultures and analysis of CCK and GLP-1 secretion. Magnesium chloride (0.0468 g/L), potassium chloride (0.34 g/L), sodium chloride (7 g/L), sodium phosphate dibasic (0.1 g/L), sodium phosphate monobasic (0.18 g/L), D-glucose (1.8 g/L) and HEPES (5.9 g/L) were used to prepare the KRB/HEPES buffer (all chemicals (reagent grade) were purchased from Sigma-Aldrich (Castle Hill, Australia)). The locations for sampling were selected based on previously published data on the small intestine morphometry of young pigs [16].

### Primary intestinal cell culture

Proximal jejunum and ileum samples were collected for primary intestinal cultures following the procedure previously described by Voortman et al. [17] to study the effect of free Lys on gut peptide secretion and the expression of AA sensors. In brief, upon arrival at the laboratory, intestinal segments were cleaned of debris using KRB/HEPES buffer, cut open longitudinally and stripped of their outer muscle layers before equally sized round samples (1.13 cm^2^) were excised using a biopsy punch (Acuderm Inc., Fort Lauderdale, USA) and placed in 24-well plates (ThermoFisher Scientific, Waltham, USA) filled with ice-cold KRB/HEPES buffer. After 30 min at room temperature, plates were moved into a humidified incubator (NU5510, Nuaire, Plymouth, USA) for 1 h at 37 °C and 5% v/v CO_2_, after which the content of each well was replaced with a new solution of prewarmed KRB/HEPES buffer containing Lys (analytical grade) (Sigma-Aldrich) at 0, 10, 20 or 30 mmol/L and no D-glucose. Plates were placed back in the incubator for an additional 1 h and then the supernatant collected, mixed with phenylmethanesulfonyl fluoride at 100 mmol/L (to prevent enzymatic degradation of gut peptides) (Sigma-Aldrich) and stored in Eppendorf tubes at −80 °C for hormone analysis. Tissue samples were placed in RNAlater (Sigma-Aldrich) at room temperature for 24 h before frozen at −80 °C for later mRNA analysis. Lactate dehydrogenase (LDH) activity was measured with Roche LDH reagent kit PLUS (4744926001 Sigma-Aldrich) in tissue samples (by evaluating the leakage of intracellular LDH into supernatant stored at 4 °C) and compared to a positive control incubated with 1% Triton-X 100 (Sigma-Aldrich), to determine the viability of the intestinal culture.

### Amino acid and hormone analysis

Blood samples were centrifuged (3,000 r/min) at 4 °C for 10 min within 1 h from collection and the plasma aliquoted into Eppendorf tubes before frozen at −80 °C until AA analysis. To prepare the plasma for the AA evaluation, samples were first centrifuged at 14,000 × *g* at room temperature for 30 min using Amicon® Ultra 0.5 mL filters (3 kDa MWCO) (Merck Millipore, Burlington, USA). The filtrate was then aliquoted into Eppendorf tubes and mixed, in the same proportion (1:1), with an internal standard solution containing 2-aminobutanoic acid and sarcosine. AA within the processed samples were then derivatised following the methodology described by Valgepea et al. [18], and the concentration analysed by using RP-HPLC. Supernatant samples were analysed for total CCK and GLP-1 using a Porcine Cholecystokinin ELISA kit from MyBioSource (MBS264395, San Diego, USA) and a glucagon-like peptide-1 (Total) ELISA kit from Merck Millipore (EZGLPT1-36 K, Burlington, USA), respectively. Intra- and inter-assay coefficients of variation for the CCK kit were 6.0% and 9.3%, respectively, whereas GLP-1 intra- and inter-assay coefficients of variation for the GLP-1 kit were 1.9% and 2.2%, respectively. Optical density recordings of the ELISA plates were performed in BMG LABTECH FLUOstar OPTIMA (BMG Labtech, Mornington, Australia).

### RNA extraction and RT-qPCR analysis

The extraction and isolation of RNA from proximal jejunum was performed using first Trizol® Reagent (15596026) and PureLink® RNA Mini kit (12183018A) (Invitrogen, Carlsbad, USA), respectively, following the manufacturer’s instruction. Sample’s RNA quality and concentration were determined using Nano Drop spectrophotometer (NanoDrop 8000, ThermoFisher Scientific, Waltham, USA). QuantiTect® Reverse Transcription kit (205313, QIAGEN, Hilden, Germany) was used for cDNA synthesis. Primers for the AA sensors and the reference genes used in this study have been previously published [5, 19, 20] and are listed in Table 2. The real-time qPCR reaction contained 5 μL of SYBR Green master mix solution, 3 μL of RNAs free water, 1 μL of cDNA sample, 0.5 μL forward and reverse PCR primers and 0.05 μL of ROX reference dye solution. The RT-qPCR program used for the analysis has been previously published [2]. In short, following initial denaturation at 95 °C for 2 min, the PCR cyclin condition was 40 cycles of 95 °C for 15 s and 60 °C for 60 s using a real time PCR system (QuantStudio 6, ThermoFisher Scientific, Waltham, USA). The housekeeping gene *GAPDH* was used as an internal control to normalize the expression of target genes. The relative expression of genes was calculated following the Pfaffl method [21].

**Table 2:** Real time PCR primers

Gene	Accession No.	Primer	Sequence (5′→3′)	Product size, bp
*CaSR*	XM_021068447.1	Forward Reverse	TGCCCAGATGACTTCTGGTCC GCACGAGATGCAGAGCACGAAGC	330
*T1R1*	XM_013988748.1	Forward Reverse	TACAACGGTCTCCTCTCGGT CAGCATGGCAAACACGTTGA	192
*T1R3*	NM_001113288.1	Forward Reverse	TGTACCAGGTTCTCGTCCCT GGCCATGAACACTAGGCTG	172
*mGluR1*	XM_005659163.3	Forward Reverse	GTCGGGAGCTCTGCTACATC GGCACTCATAAACCTGGGCT	230
*mGluR4*	XM_001928369.6	Forward Reverse	GCCCAAGGTCTACGTCATCC CCTGGCTAGATCGCATGGTT	228
*SLC7A1*	NM_001012613.1	Forward Reverse	TCTGGTCCTGGGCTTCATAA ACCTTCGTGGCATTGTTCAG	123
*SLC7A2*	NM_001110420.1	Forward Reverse	GCAACAACTGGCGAAGAAGT GGCATCATAAGGGTCAAAGC	122
*GAPDH*	NM_001206359.1	Forward Reverse	TGGTGAAGGTCGGAGTGAAC GAAGGGGTCATTGATGGCGA	104

### Statistical analysis

Statistical analysis was performed using R software (RStudio, Inc., Boston, Massachusetts, USA). All data are expressed as the mean ± SEM. Performance parameters [average daily feed intake (ADFI), average daily gain (ADG), and gain to feed ratio (G:F)] were analysed using a linear mixed model with “sex”, “weight”, “Lys dietary dose”, “week”, and the interaction of the last 2 as fixed effects, and “pig” as random effect. Plasma AA concentration from T0 and T3 pigs were analysed with a linear mixed model with “Lys dietary dose”, “sex” and “weight” as fixed effect and “pig” as random effect. Ex vivo data (gut hormone secretion and AA sensors gene expression) were analysed with a linear mixed model considering “Lys mmol/L treatment” as fixed effect and “pig” as random effect. Comparison of the treatments vs. the control were performed with a Dunnett test. The number of samples (*n*) refers to the number of pigs used. Results were considered statistically significant at *P* ≤ 0.05 and trends when 0.05 ≤ *P* ≤ 0.10.

## Results

The ADFI, ADG and G:F conversion ratio of post weaning pigs for the 4 dietary treatments are shown in Table 3. No significant differences were observed during the first 2 weeks post weaning. ADFI was linearly reduced (*P* < 0.001) with the increase of dietary Lys (T0 > T1 > T2 > T3) during the third week post weaning. The decrease in ADFI during the third week accounted for approximately 1.5 g for each percentage (as nutrient content) of SID Lys above the level of the control diet (up to approximately 150 g reduced feed intake at 200% of SID Lys requirement). When looking at the overall (0 to 21 d post weaning) effect of dietary Lys content on ADFI, higher levels of Lys in the feed tended (*P* < 0.10) to reduce feed intake in post weaning pigs by approximately 0.8 g for each percentage (as nutrient content) of SID Lys added above the level of the control diet (up to approximately 80 g of reduced feed intake at 200% of SID Lys requirement). However, no significant linear response was observed for ADG during the first, second, third week or the overall period. A linear response of Lys levels to reduce G:F ratio was found as a trend (*P* < 0.10) during the first week. Nonetheless, no differences were observed during the second week, third week or the overall period. The quadratic effect of excess dietary Lys content on performance parameters was not significant (thus, results were not included).

**Table 3:** Performance of post weaning pigs fed dietary Lys excesses

Performance	Dietary treatments^1^				SEM	*P*-value^2^
	T0	T1	T2	T3		
ADFI^3^, g/d						
Week 1	222.02	232.74	212.14	184.98	0.458	0.370
Week 2	472.86	446.43	425.00	424.40	0.458	0.353
Week 3	760.24	692.02	657.29	599.64	0.458	0.003
Overall	485.04	457.06	431.48	403.01	0.374	0.055
ADG^4^, g/d						
Week 1	156.90	158.69	148.43	109.95	0.443	0.276
Week 2	335.36	303.69	296.29	287.86	0.443	0.374
Week 3	532.14	487.38	498.43	488.09	0.443	0.282
Overall	341.47	316.59	314.38	295.30	0.304	0.155
G:F ratio^5^						
Week 1	0.70	0.70	0.70	0.56	0.00079	0.078
Week 2	0.68	0.67	0.69	0.68	0.00079	0.938
Week 3	0.72	0.71	0.76	0.81	0.00079	0.245
Overall	0.70	0.69	0.71	0.68	0.00048	0.771

^1^*T0* 100% Lys requirement, *T1* 120% Lys requirement, *T2* 150% Lys requirement, *T3* 200% Lys requirement

^2^Linear contrast on the effect of Lys dietary content on ADFI, ADG and G:F ratio

^3^Average daily feed intake

^4^Average daily gain

^5^Gain to feed ratio

**Fig. 1:**
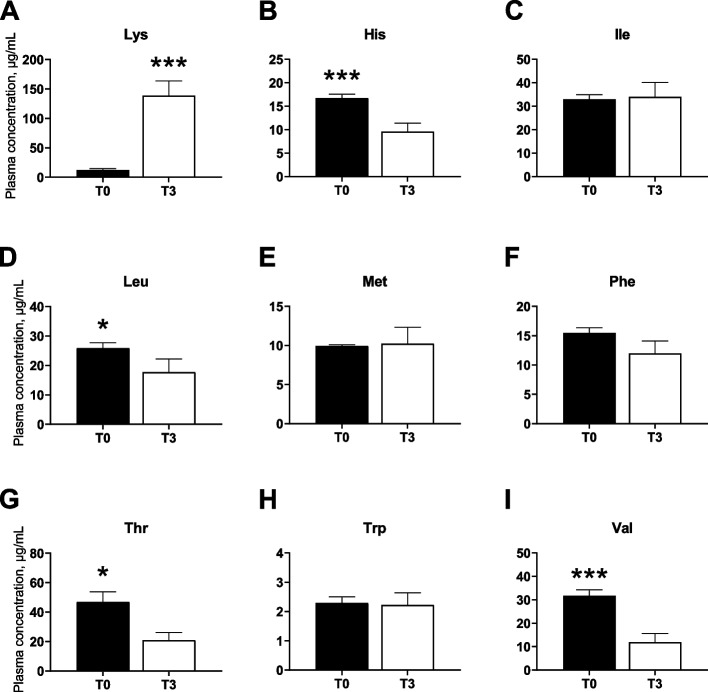
Blood levels of essential amino acids (EAA) in pigs fed Lys dietary excesses. Effect of dietary Lys levels (100% of requirements (T0) vs. 200% of requirements (T3)) on EAA (Lys (**A**), His (**B**), Ile (**C**), Leu (**D**), Met (**E**), Phev(**F**), Thr (**G**), Trp (**H**) and Val (**I**)) plasma levels in young pigs. Data are presented as the mean ± SEM (*n* = 6). ^***^P ≤ 0.001, ^**^P ≤ 0.01, ^*^P ≤ 0.05

**Fig. 2:**
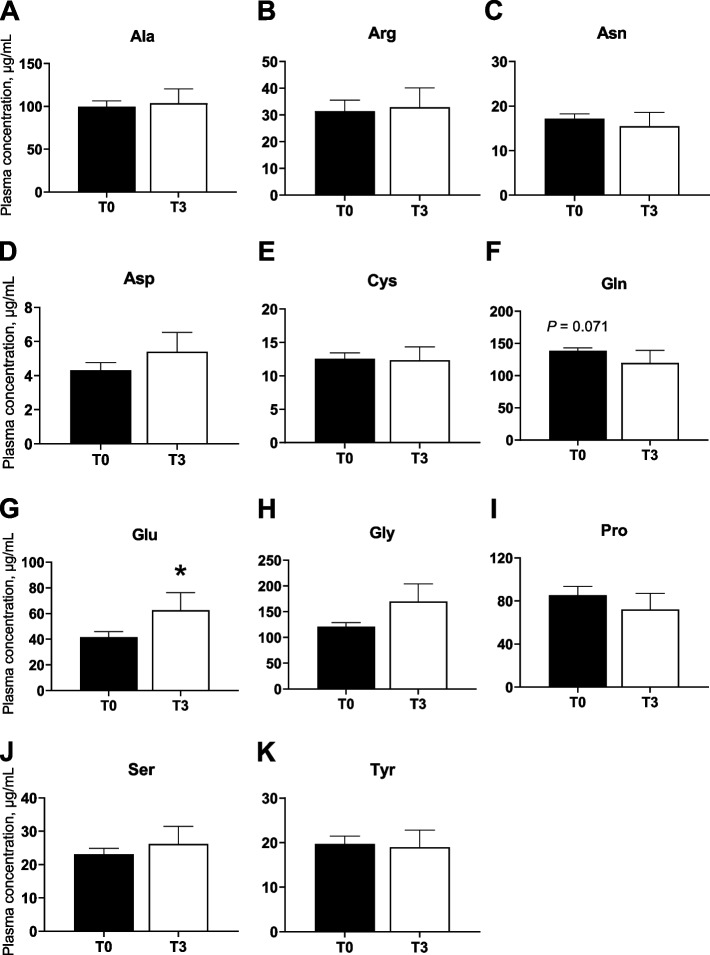
Blood levels of non-essential amino acids (NEAA) in pigs fed Lys dietary excesses. Effect of dietary Lys levels (100% of requirements (T0) vs. 200% of requirements (T3)) on NEAA (Ala (**A**), Arg (**B**), Asn (**C**), Asp (**D**), Cys (**E**), Gln (**F**), Glu (**G**), Gly (**H**), Pro (**I**), Ser (**J**) and Tyr (**K**)) plasma levels in young pigs. Data are presented as the mean ± SEM (*n* = 6). ^*^P ≤ 0.05, tendency = 0.05 ≤ *P* ≤ 0.10

The circulating levels of plasma EAA and NEAA in T0 or T3 fed pigs are shown in Fig. 1 and 2, respectively. Plasma Lys concentrations increased by more than 10-fold (*P* < 0.001) in the T3 compared to T0. In contrast, plasma levels of His and Val (*P* < 0.001) as well as Thr and Leu (*P* < 0.05) were significantly lower in T3 than T0 fed pigs. Of the NEAA, plasma Glu increased (*P* < 0.05) while Gln tended (*P* = 0.07) to decrease in T3 compared to T0 pigs. Non-significant differences were observed for other AA plasma concentrations between the dietary treatments. Overall, total EAA levels in plasma, but not NEAA, were significantly higher in T3 when compared to the T0 group (*P* < 0.001).

**Fig. 3:**
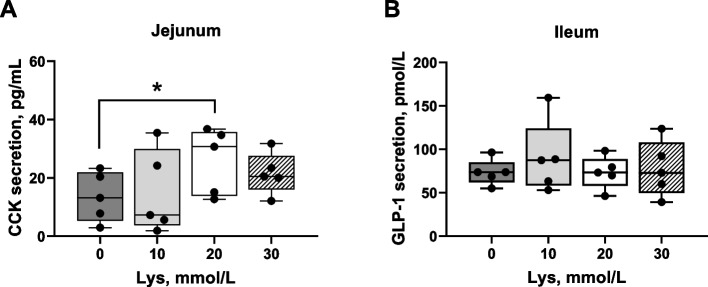
Cholecystokinin (CCK) and glucagon-like peptide 1 (GLP-1) concentration in porcine intestinal cultures exposed to Lys. CCK and GLP-1 secretion using an ex vivo jejunum (**A**) or ileum (**B**) culture from young pigs. Tissue cultures were incubated for 1 h with a KRB/HEPES buffer containing 0 (control), 10, 20 and 30 mmol/L Lys. Data are presented as the mean ± SEM of 3 biological replicates per pig (*n* = 5). ^*^P ≤ 0.05

**Fig. 4:**
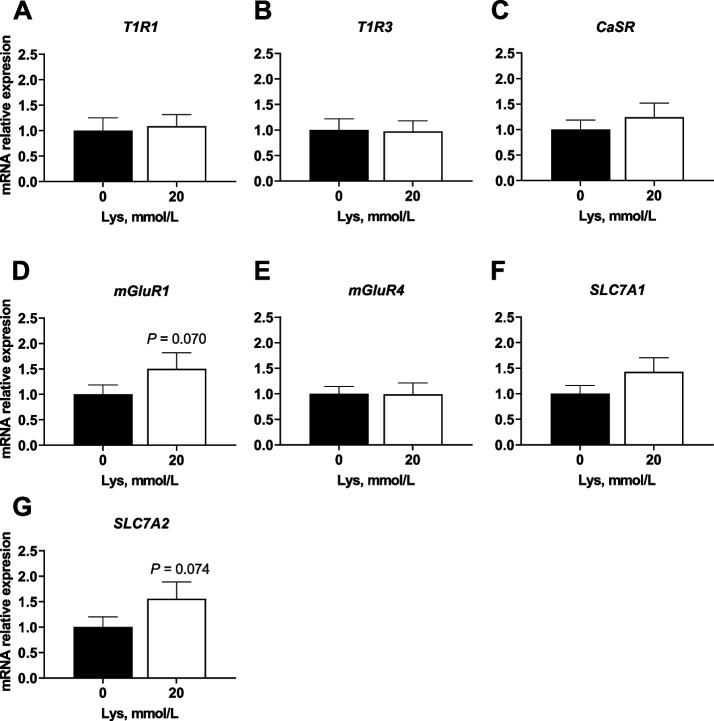
Gene expression of AA sensors in porcine jejunum exposed to Lys. Gene expression (mRNA abundance) of AA receptors and transporters (*T1R1* (**A**), *T1R3* (**B**), *CaSR* (**C**), *mGluR1* (**D**), *mGluR4* (**E**), *SLC7A1* (**F**) and *SLC7A2* (**G**)) in the proximal jejunum of young pigs following 1 h incubation with a KRB/HEPES buffer containing 0 (control) or 20 mmol/L Lys. Data are presented as mean ± SEM (*n* = 6). *P* values indicate a statistical trend: 0.05 ≤ *P* ≤ 0.10

Levels of LDH were 6.77% ± 2.15% and 5.50% ± 1.93% for proximal jejunum and ileum, respectively, when compared to the positive control. Thus, based on the LDH results, Lys at 10, 20 or 30 mmol/L had no significant impact on the integrity of the intestinal tissues (below 10% of the positive control denotes good viability). Gut hormone concentration following Lys exposure are shown in Fig. 3. CCK secretion was enhanced in proximal jejunum tissues incubated with 20 mmol/L (*P* < 0.05) of Lys compared to the control (KRB/HEPES buffer free of Lys). In contrast, GLP-1 levels did not significantly change in the ileum following exposure to 10, 20 or 30 mmol/L Lys when compared to the control. The effect of Lys at 20 mmol/L (dose chosen for analysis based on gut hormone results) on the mRNA expression levels of AA sensors and receptors in proximal jejunum are illustrated in Fig. 4. The metabotropic glutamate receptor 1 (*mGluR1*) and the cationic AA transporter solute carrier family 7 member 2 (*SLC7A2*) gene expression level tended (*P* = 0.07) to increase in 20 mmol/L Lys treated tissues compared to the control group. Besides these 2 AA sensors, Lys at 20 mmol/L did not enhance the gene expression of other AA receptors and transporters tested in this study.

## Discussion

This study illustrated the effect of excess dietary Lys on performance and satiety biomarkers in post weaning pigs. It was hypothesized that Lys dietary excess would significantly reduce feed intake, stimulate the secretion of CCK in the gastrointestinal tract and alter the concentration of EAA and NEAA in blood. In general, the hypothesis was supported by the results.

As anticipated, the dietary inclusion of Ala, particularly in the control diet, did not have a negative effect on feed intake and growth in pigs, which allowed us to differentiate the satiating effect of increasing dietary Lys compared to nitrogen contents. A linear effect of dietary Lys levels on ADFI was observed indicating that the higher the Lys excess the more feed intake was inhibited in post weaning pigs. These results are consistent with the study of Edmonds and Baker [22] in which a depression in feed intake and weight gain was observed in pigs fed dietary increments of Lys between 1.15% and 4.6% (as nutrient content). In the present study, the excess of Lys did not result in changes in growth but marginally decreased the G:F ratio reflecting that the catabolism of excess Lys (nitrogen clearance from the system) could have reduced the energy available for growth (protein accretion) as the liver and kidneys, which are major sites of amino acid degradation, will require more energy to metabolize the high amounts of circulating Lys [23]. However, the same may not have occurred in the pigs supplemented with higher quantities of Ala (control diet) as a significant portion of this NEAA is generally oxidised and used as metabolic fuel by enterocytes before reaching the blood stream in pigs [24]. The absence of statistical significance observed for some of the performance parameters with differences above 10% among treatments (e.g., ADG and G:F ratio) may be related to the relative low number of pigs used (*n* = 6/treatment).

Consistent with the anorexigenic effect observed in the animals, a 20 mmol/L Lys solution increased CCK secretion from the proximal jejunum. In pigs, AA concentrations above 10 mmol/L have been described in the ileum following consumption of protein meals [25]. This indicates that the doses of Lys tested in the present study, and their impact on CCK, are physiologically relevant. Moreover, these results agree with previous studies indicating an effect of Lys on CCK plasma levels in pigs after an oral gavage [4]. The ability of Lys to stimulate CCK secretion in the jejunum, but not the duodenum, may be related to the higher expression of AA sensors in this segment of the small intestine since it is the principal site of AA absorption [2, 20]. Compatible with this observation, it was observed that the expression of *mGluR1* and *SLC7A2* tended to increase in Lys treated jejunal tissues. Both *mGluRs* and AA transporters are expressed in enteroendocrine cells [26, 27]. However, their potential involvement in CCK secretion is speculative since gut hormone release and AA sensing has not been described in pigs to date [28, 29].

In contrast to CCK, Lys did not trigger the release of GLP-1 in the ileum at even supraphysiological doses (30 mmol/L) suggesting that the Lys anorexigenic effect may be independent of enteroendocrine L-cell activation in pigs. It is important to mention as well that, in this study, it is unlikely that high doses of free Lys could have reach the distal ileum and triggered the release of GLP-1 as crystalline AA true ileal digestibility has been reported to be close to 100% in pigs (their absorption is more proximal to that of protein bound AA) [30].

Beside gut peptides, post absorptive signals related to changes in the concentration of AA in blood may also be interpreted as key peripheral signals relevant to satiation and satiety [6, 7, 31]. For instance, intravascular injection of Lys reduced feed intake to a similar extent to an oral gavage in mice, suggesting a direct effect of the AA in the CNS [8]. In our study, the provision of excess Lys in the feed altered the AA blood profile of pigs in 4 distinct patterns. Firstly, Lys supplementation significantly increased the concentration of circulating Lys (pattern 1). These results agree with previous studies in finishing and growing pigs illustrating a linear increase in Lys plasma concentrations with increasing dietary Lys doses [12, 13, 32]. This linear increase has been attributed to the low catabolism and oxidative properties of Lys, which may contribute to a substantial amount of the AA reaching the blood stream intact from the gastrointestinal tract [33]. Once in the blood, Lys may interact with the hypothalamus to modulate appetite in pigs. The interaction of blood AA with the CNS may be partially mediated by tanycytes, radial glial-like cells that line the third ventricle (funnel-shaped structure that is in contact with the hypothalamus) and have access to circulating plasma due to their proximity to fenestrated capillaries [34]. Lys, in particular, has been identified as a robust tanycyte stimulant in rodents [35]. Thus, Lys may interact with the hypothalamus inducing satiety through the stimulation of tanycytes in pigs as well. Future research should investigate the effect of intravenous injections of Lys on feed intake in pigs to corroborate a satiating effect independent of gut hormone secretion.

Secondly, Lys supplementation also affected the circulating levels of five additional EAA and two NEAA. While Glu plasma levels increased (pattern 2), Leu, Val, Thr, His (pattern 3), and Gln levels (pattern 4) decreased in the Lys supplemented pigs compared to the control group. Increases in Glu plasma levels have been associated with low feed intake and satiety in rodents [8]. Interestingly, in previous studies, pigs fed excess Lys showed higher Glu circulating blood levels [13]. The primary site of Lys catabolism is in the liver, producing Glu as one of the main metabolites [33, 36]. Thus, dietary Lys excess seems to result in an increase of liver catabolic pathways raising the circulating levels of Glu. Hence, the CNS sensing of increased Glu plasma levels may contribute to the anorexigenic effect of dietary Lys excess in pigs.

On another note, Lys supplementation was associated with decreased circulating levels of Thr, His and Val. These results are consistent with observations previously reported in growing and finishing pigs [13, 32]. In contrast, Lys supplementation did not affect Leu levels in our experiment. The low blood levels of Leu observed following the increase in Lys intake may be explained by an increase in the activation of intestinal AA antiporters that facilitate the uptake of extracellular cationic AA in exchange for intracellular Leu [37]. The effect of excess dietary Lys on Leu plasma levels is of particular interest considering the strong appetite modulatory properties of Leu in pigs [10, 11]. High circulating levels of Leu may reduce feed intake by interacting with the key hypothalamic (mediobasal and paraventricular hypothalamus) and brainstem structures (nucleus of the solitary tract) as shown in mice [38, 39]. The opposite effect would be expected if plasma levels of Leu were kept low linked to Lys excess levels. The gastrointestinal interaction between Leu and Lys is a subject that merits further investigation.

In contrast to Leu, a direct effect of plasma Val and His on appetite modulatory structures within the hypothalamus has not been established. However, previous studies reported lower performance following the provision of Val and His deficient diets in pigs [40–42]. We speculate that the reduction in plasma levels of His and other AA may have been sensed by the brain contributing to the reduced feed intake observed in Lys over supplemented pigs [43]. Potentially, the decrease in plasma levels of EAA are a consequence of a rise in protein synthesis in the whole body related to the increased availability of Lys [13].

## Conclusions

Excess dietary Lys reduced feed intake by enhancing satiety in post weaning pigs. Increasing Lys levels in starter diets raised plasma Lys and Glu while lowering circulating levels of Leu, His, Val, and Thr. In addition, Lys increased CCK secretion in the proximal jejunum. Dietary excesses of Lys should be narrowed, when possible, to avoid any potential negative effects on appetite in post weaning pigs.

## Data Availability

All data generated or analysed during this study are available from the corresponding author upon reasonable request.

## Abbreviations

AA: Amino acid
ADFI: Average daily feed intake
ADG: Average daily gain
BCAA: Branched-chain amino acid
CCK: Cholecystokinin
CNS: Central nervous system
EAA: Essential amino acid
G:F: Gain to feed ratio
GLP-1: Glucagon-like peptide 1
LDH: Lactate dehydrogenase
mGluR1: Metabotropic glutamate receptor 1
NEAA: Non-essential amino acid
SID: Standard ileal digestible
SLC7A2: Solute carrier family 7 member 2
